# A Mobile Health Intervention for Adolescents Exposed to Secondhand Smoke: Pilot Feasibility and Efficacy Study

**DOI:** 10.2196/18583

**Published:** 2020-08-19

**Authors:** Natalie Nardone, Jeremy Giberson, Judith J Prochaska, Shonul Jain, Neal L Benowitz

**Affiliations:** 1 Clinical Pharmacology Research Program, Division of Cardiology Department of Medicine University of California San Francisco, CA United States; 2 Stanford Prevention Research Center Department of Medicine Stanford University Stanford, CA United States; 3 Department of Pediatrics Zuckerberg San Francisco General Hospital University of California San Francisco, CA United States; 4 Departments of Medicine University of California San Francisco, CA United States; 5 Department of Bioengineering and Therapeutic Sciences University of California San Francisco, CA United States

**Keywords:** secondhand smoke, adolescents, cotinine, mHealth, intervention

## Abstract

**Background:**

Secondhand smoke (SHS) exposure in children and adolescents has adverse health effects. For adolescents of lower socioeconomic status (SES), exposure is widespread, evidenced in the measurement of urinary cotinine, a major metabolite of nicotine. Direct intervention with exposed children has been proposed as a novel method, yet there is minimal evidence of its efficacy. Combining this approach with a mobile health (mHealth) intervention may be more time and cost-effective and feasible for adolescent populations.

**Objective:**

In this pilot study, we assessed the feasibility and preliminary evidence of efficacy of a 30-day text message–based mHealth intervention targeted at reducing SHS exposure in adolescent populations of low SES.

**Methods:**

For the study, 14 nonsmoking and nonvaping participants between the ages of 12-21 years exposed to SHS were enrolled. The intervention consisted of a daily text message sent to the participants over the course of a month. Text message types included facts and information about SHS, behavioral methods for SHS avoidance, or true-or-false questions. Participants were asked to respond to each message within 24 hours as confirmation of receipt. Feasibility outcomes included completion of the 30-day intervention, receiving and responding to text messages, and feedback on the messages. Efficacy outcomes included a reduction in urinary cotinine, accuracy of true-or-false responses, and participants’ perceptions of effectiveness.

**Results:**

Of the 14 participants that were enrolled, 13 completed the intervention. Though not required, all participants had their own cell phones with unlimited text messaging plans. Of the total number of text messages sent to the 13 completers, 91% (372/407) of them received on-time responses. Participant feedback was generally positive, with most requesting more informational and true-or-false questions. In terms of efficacy, 54% (6/11) of participants reduced their cotinine levels (however, change for the group overall was not statistically significant (*P*=.33) and 45% (5/11) of participants increased their cotinine levels. Of the total number of true-or-false questions sent across all completers, 77% (56/73) were answered correctly. Participants’ ratings of message effectiveness averaged 85 on a scale of 100.

**Conclusions:**

In this pilot study, the intervention was feasible as the majority of participants had access to a cell phone, completed the study, and engaged by responding to the messages. The efficacy of the study requires further replication, as only half of the participants reduced their cotinine levels. However, participants answered the majority of true-or-false questions accurately and reported that the messages were helpful.

## Introduction

Secondhand smoke (SHS) exposure is associated with adverse health effects in children and adolescents, including respiratory disease and asthma [1-4]. In a study of adolescents of low socioeconomic status (SES) in San Francisco, California, 76% were found to have recent light or heavy SHS exposure based on biochemical screening of the major nicotine metabolite, cotinine, with ranges of 0.05-30 ng/mL [5]. In a follow-up study, these adolescents reported common exposure to SHS in public areas; however, reported SHS in homes and cars significantly predicted biochemical exposure [6]. Interventions targeted at reducing SHS may improve adolescent health.

Past interventions to reduce SHS exposure in children and adolescents have demonstrated mixed results. Many have focused on intervening on parental or caregiver smoking status, with a recent meta-analytic review demonstrating that some studies were effective at reducing SHS exposure while many others were not [7]. In addition, the focus on caregivers may not translate to patient care, as pediatricians are often reluctant to intervene on parental smoking [8].

Intervening directly with exposed children without the inclusion of cessation counseling for parents is a novel approach [9,10]. One proposed study for decreasing SHS exposure describes an educational intervention developed from behavioral change theory, consisting of three 40-minute educational sessions on the adverse effects of tobacco, international laws regarding tobacco, and methods to prevent tobacco exposure; weekly take-home brochures; and 3 reinforcement lessons [9]. A school-based study conducted in Bangladesh with children aged 10-12 years evaluated a similar method, consisting of two 45-minute educational sessions over a 2-day period followed by four 15-minute refresher sessions over the following month; saliva cotinine at the 2-month follow-up showed a significant difference between the intervention (0.53 ng/mL) and control groups (2.02 ng/mL) [10].

Delivery of SHS prevention educational interventions for youth via mobile health (mHealth) technologies may be more time and cost-effective and may be particularly acceptable to an adolescent population. MHealth interventions, such as sending daily text messages, have been developed utilizing behavioral [11] and social cognitive theories [12], and they have been effective for increasing physical activity among adolescents with attention deficit hyperactivity disorder (ADHD) [13], for promoting weight loss in adolescents with obesity [14], and for type 1 diabetes management in children and adolescents, with a focus on healthy eating and activity [11].

While mHealth interventions have been efficacious for a variety of indications [9,10,13], a downside to these methods may be low levels of engagement. Studies have provided participants with cell phones or prepaid phone cards to facilitate their participation [12,15]. Others have reported that technical problems led to a loss of data and affected the participants’ levels of motivation to continue in the study [15-18]. Past studies have assessed feasibility as participant satisfaction and rates of participant retention [15,16]. In determining the efficacy of mHealth interventions, outcome measures are varied and include biomarker reductions, self-reported efficacy, and changes in disease or condition knowledge [11,13,14]. To date, mHealth interventions have not been developed to assist adolescents in reducing SHS exposure.

In this pilot study, we examined the feasibility and efficacy of a novel mHealth intervention to reduce measured SHS exposure in a small sample of urban adolescents of low SES. Feasibility was measured by intervention completion, receipt of text messages, participant responses to messages, and participant feedback on the quality and delivery of the messages. Efficacy was measured by reduction in urinary cotinine, SHS knowledge, and participant perception of the effectiveness of the messages for changing their behavior to reduce SHS exposures.

## Methods

### Participants

Participants of the ages of 12-21 years were recruited from July 2017 to May 2018. Recruitment occurred through (1) invitation from prior research studies [5,6], if the individual’s cotinine level was within the heavy SHS range (urinary cotinine 0.25-30 ng/mL) during their prior study involvement; (2) social media ads on Facebook and Instagram; and (3) flyers posted in the Children’s Health Center (CHC) at the Zuckerberg San Francisco General Hospital. We included participants over the age of 18 years, as the CHC services adolescent patients up to the age of 21 years. A screening measure assessed tobacco use and SHS exposure [6]. Self-reported active use of cigarettes, electronic cigarettes, or other tobacco products was an exclusion.

The University of California, San Francisco (UCSF), Institutional Review Board approved the research. Informed consent was obtained from the youths and from parents of those under the age of 18 years. Parents were asked if their adolescents had their own cell phones with unlimited text messaging services. The study team was prepared to provide a study phone on loan and gift cards for messaging fees, if applicable.

### Measures

At baseline and the 30-day follow-up, the adolescents provided a urine sample for cotinine testing, and they self-reported their exposure to SHS across several environments for the past 7 days and the past 24 hours [6]. At the follow-up visit, through an online questionnaire, the adolescents were asked about their experiences with the intervention, with questions like “Have the text messages made you more active in reducing your contact with secondhand smoke?” They were also asked to rate how helpful the messages were (on a scale of 0-100), how they felt about receiving them, if they shared the messages with others, what they liked most about the study, how the study could be improved, and why they may not have responded to the messages. Adolescents’ age, sex, and race were recorded, and nicotine and tobacco product ever use were assessed. All questionnaires were created for this study using standard items where available.

### Text Message Intervention

The intervention consisted of one text message sent per day via Outlook’s SMS text message service, a free feature available as part of the Outlook package (version 16.0; Microsoft Corp). Messages were cued in Outlook to be sent from the research center’s email address to the participants’ phones at a time of their choosing. Participants were asked to respond to each message within 24 hours as confirmation of receipt. Text message categories included facts and information about SHS (eg, “Breathing secondhand smoke for a short period of time can hurt your body”), behavioral methods to avoid SHS exposure (eg, “Avoid SHS today by walking away from someone who is smoking”), and “bonus” true-or-false questions (eg, “True or False: SHS is annoying, but it’s not really a health concern”). All messages included emojis, which were pretested to ensure compatibility with differing phone models. A total of 35 text messages were created to ensure that there were enough for additional days if participants were not able to return for their follow-up visit on day 30. A 30-day duration was selected as it was similar to prior interventions designed for an adolescent population [9,10]; in addition, 30 days is long enough to see a change in steady-state cotinine levels [19].

The primary sources for the information presented in the messages were websites from government agencies (http://cdc.gov/, http://smokefree.gov, http://cancer.gov) and the 2006 Surgeon General Report [1].

### Analytical Chemistry

Baseline and follow-up urine samples were analyzed for cotinine by liquid chromatography–tandem mass spectrometry [20]. Cotinine, the main proximate metabolite of nicotine, has a half-life of about 16 hours [19] and is a biomarker of ongoing or recent exposure (past 5-6 days). The limit of quantitation (LOQ) for cotinine was 0.05 ng/mL. Cotinine cutpoints were 0.05-30 ng/mL for light or heavy SHS and >30 ng/mL for active smoking or vaping [5].

### Statistical Analysis

We utilized descriptive statistics to summarize participant characteristics and rates of response to text messages. To assess changes in cotinine levels pre- and postintervention, we utilized a Wilcoxon signed rank test, a nonparametric equivalent to a paired sample *t* test. Biomarkers falling below the limit of quantitation (BLQ) were replaced with the LOQ divided by the square root of 2.

## Results

### Demographics and SHS Exposure at Baseline

A total of 263 participants were screened, and 14 consented and were enrolled in the study. The primary reason for ineligibility was not meeting the criteria for recent SHS exposure (43%, 114/263); other individuals were ineligible for age (13%, 34/263), no longer lived in the area (3%, 8/263), or were not interested in participating (29%, 77/263). Some were eligible and scheduled for a baseline visit but did not arrive (6%, 16/263).

Of the 14 enrolled participants, 3 (21%) were male adolescents and 11 (79%) were female adolescents; when asked to self-report race/ethnicity, 3 (21%) were Hispanic, 2 (15%) were non-Hispanic White, 3 (21%) were Black, 1 (7%) was Asian, and 5 (36%) were multiracial. Participants’ ages ranged from 12 to 20 years, with a mean of 17 (SD 2.39) years. All participants reported SHS exposure within the last 7 days, with the most highly reported environments being public areas (30%), residences (30%), and cars (21%).

**Figure 1:**
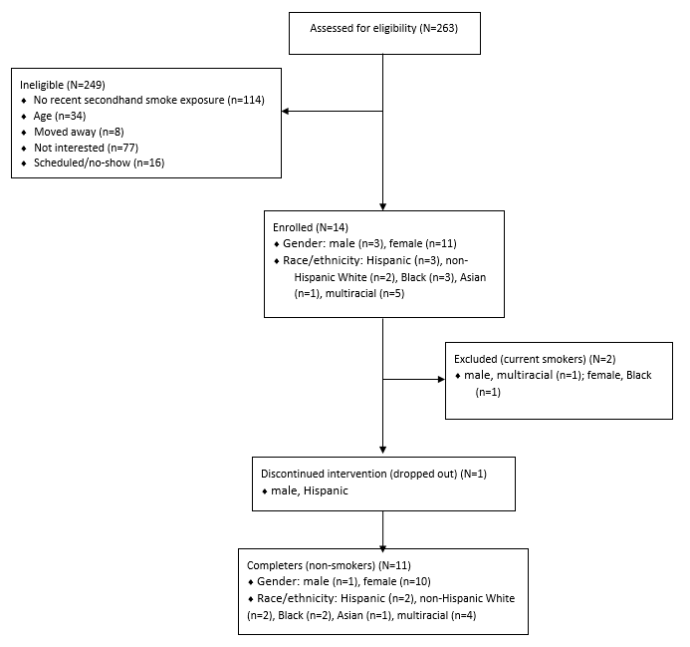
Consolidated standards of reporting trials (CONSORT) flow diagram for participants recruited (N=263) and enrolled (n=14).

Nicotine and other tobacco product use was reported as ever use of cigarettes (50%, 7/14), blunts (64%, 9/14), electronic cigarettes (43%, 6/14), pipes (36%, 5/14), cigars or cigarillos (21%, 3/14), or hookah (36%, 5/14). Figure 1 shows the consolidated standards of reporting trials (CONSORT) flow diagram.

### Intervention Feasibility

Of the 14 participants enrolled, 13 (93%) completed the intervention, as 1 participant dropped out midstudy. The remaining analyses focus on the 13 adolescents who completed the intervention (completers).

Due to variability in the scheduling of the follow-up visits and occasional missed messages due to Outlook shutdowns during routine software updates, the adolescents received a range of 19 to 34 text messages (mean 31.3, SD 7.71). On average, 91% (SD 12.61; range 55%-100%) of the messages received a reply from the participants within 24 hours of sending.

Participant feedback indicated that participants generally liked receiving the messages and would have wanted to see more, especially from the informational and true-or-false categories (Table 1). The majority (12/13) shared the messages with friends and family. One participant reported having technical difficulties.

**Table 1:** Participant feedback from the intervention completers (n=13).

Question & Response Options		Participant Response Selections	
**How have the text messages made you more active in reducing your contact with secondhand smoke? n (%)**			
	I have avoided people smoking around me.		11 (85)
	I noticed more people smoking around me.		8 (62)
	I asked friends not to smoke around me.		4 (31)
	I stopped smoking myself.		1 (8)
	I wasn’t active in reducing my contact.		1 (8)
On a scale of 0-100 (0=not helpful, 100=very helpful), how would you rate the text messages in helping you remember the dangers of being in contact with secondhand smoke? Mean (SD)		85.8 (23)	
On a scale from 0-100 (0=too many to read, 100=I would have liked more), how did you feel about getting the text messages? Mean (SD)		85.2 (20.2)	
**Did you talk to anyone else about the information in the text messages? n (%)**			
	I shared with both family and friends.		5 (38)
	I shared with family only.		4 (31)
	I share with friends only.		3 (23)
	I didn’t share any messages.		1 (8)
**What did you like most about this study? n (%)**			
	Getting gift cards		12 (92)
	Receiving text messages		9 (69)
**What suggestions do you have for us to make this study better**? **n (%)**			
	Nothing		8 (62)
	More true-or-false questions		2 (15)
	More information on side effects of secondhand smoke		1 (8)
	More text messages		1 (8)
	Make sure there are no technical issues		1 (8)
**Please let us know why you may have not responded to our messages; n (%)**			
	I didn’t have my cell phone with me.		5 (38)
	My phone service changed.		3 (23)
	No particular reason.		3 (23)
	I was too busy.		2 (15)

### Intervention Efficacy

At baseline, despite denying recent tobacco use on the screener, 15% (2/13) of the participants had cotinine levels indicative of active smoking or vaping (283.1 ng/mL and 31.0 ng/mL). Further, 46% (6/13) of the participants had cotinine levels indicative of high SHS exposure (0.25-30 ng/mL), 31% (4/13) had cotinine levels indicative of low SHS exposure (range 0.05-0.25 ng/mL), and 8% (1/13) had BLQ cotinine levels. All further cotinine results reflect only the 11 completers of the intervention who were nonsmokers at baseline.

The geometric mean cotinine levels for the 11 completers were 0.48 ng/mL at baseline and 0.83 ng/mL at follow-up, including 1 participant who was nearing active smoking levels (cotinine=29.42 ng/mL) and 1 participant who was BLQ (cotinine=0.04 ng/mL). With the BLQ participant and the near-active smoking levels participant excluded, the geometric mean cotinine levels were 0.67 ng/mL at baseline and 0.09 ng/mL at follow-up.

**Figure 2:**
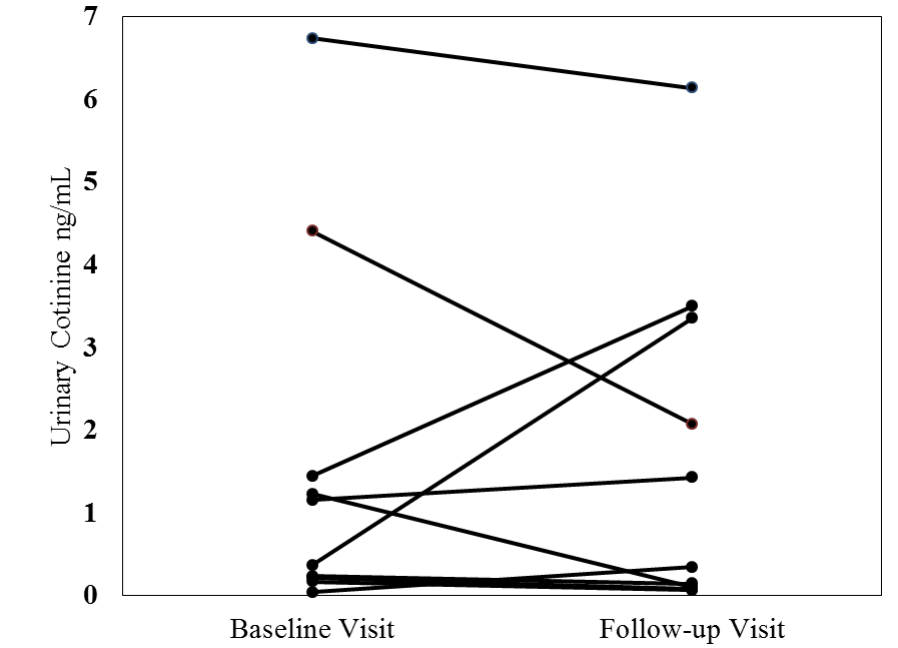
Urinary cotinine levels pre- and postintervention for nonsmoking intervention completers (n=9).

Of the 11 completers, 5 had an increase in cotinine and 6 reduced their cotinine levels; 4 participants reduced their levels by >50%. The Wilcoxon signed rank test indicated no significant difference for the entire sample (*P*=.66). The 2 participants excluded as active smokers at baseline had cotinine levels consistent with active smoking at follow-up (473.8 and 43.8 ng/mL). Cotinine levels pre- and postintervention are shown in Figure 2, excluding the participant with near-active smoking levels and the participant who was BLQ at follow-up.

As a reflection of the gains made by participants in SHS knowledge, the accuracy of the true-or-false responses averaged 77% (SD 28; range 40% - 100%) across participants. Almost all the participants (12/13, 92%) perceived the messages to be helpful in reducing their SHS exposure. Avoiding people smoking was the most commonly endorsed strategy participants used to limit their SHS exposure. One participant said the messages did not encourage them to be active in reducing their SHS (Table 1).

## Discussion

### Principal Findings

In this pilot study, we sought to determine the feasibility and preliminary effectiveness of a 30-day mHealth intervention for adolescents to avoid SHS exposure. This was a novel approach to help adolescents limit their exposure to SHS, as we sought to intervene directly with the nonsmoking, SHS-exposed youth rather than their caregivers.

Our results support that mHealth interventions are feasible in an adolescent sample. Most participants completed the study, and all had technology accessible to them to receive the text messages. Nearly all participants remained in the study for the 30-day period. There was a high rate of response to the messages, and if participants did not respond, it was mostly due to not having their phones at the time the message was sent. Participants reported that they would have liked to see more messages, especially in the information and true-or-false categories.

The efficacy findings are equivocal. Approximately half of the sample reduced their cotinine levels while the other half increased. With many participants starting in the low range of SHS exposure, a floor effect may have occurred in that the lower limit did not allow for a significant change to be exhibited. The increased biochemical exposure may reflect that the intervention was not successful in impacting specific types of exposures, or the timing of exposures in conjunction with the baseline and follow-up urine collections could have varied. Participants scored well on the true-or-false messages, indicating they were reading and processing the information. The majority of participants rated the messages as effective in changing their behavior to avoid SHS; however, these behavioral changes did not result in a significant cotinine reduction for the sample overall.

### Limitations

The limitations of this pilot study included a small and mostly female sample, a generally low level of SHS exposure, and recent tobacco use in 2 of the participants at baseline despite screening procedures. In addition, our study was conducted in a single geographic setting with progressive clean air laws but unavoidable SHS exposure in the urban environment. Although all participants reported exposure to SHS in the past 7 days, 1 (8%) participant did not meet the cotinine criteria for biochemical exposure.

Although the results of this study support the feasibility of conducting an mHealth intervention in adolescents, we cannot generalize the results to a broader population due to the small sample size. Moreover, we had only one follow-up visit at the end of the 30-day period, which did not allow us to evaluate the long-term effects of this intervention.

Due to time constraints on performing the assays, we were unable to analyze baseline cotinine levels prior to entrance in the study. Our study experienced periodic technical difficulties with occasional Outlook updates delaying or eliminating the deployment of text messages. We did not ask about nicotine or tobacco product use at the follow-up visit, so we cannot determine if some increases in cotinine were due to SHS exposure or to one’s personal product use. Additionally, a few of our participants were over the age of 18 and may reflect more of a young adult population, with different levels of access to nicotine and tobacco products. At the time this study was conducted, the minimum tobacco sales age in California was 21 years, and all study participants were aged 20 years and younger.

### Suggestions for Future Research

Researchers utilizing mHealth interventions in adolescent populations should consider providing interactive and informational messages. In addition, they should consider utilizing a follow-up period longer than 30 days to evaluate if there are long-term effects of the intervention. If applying this intervention strategy to limit SHS exposure, researchers should utilize an expired carbon monoxide monitor to exclude active tobacco users, or a dipstick cotinine test for active electronic cigarette users during the screening process. Researchers should ensure a reliable method for text message transmissions. Technical issues occurred utilizing Outlook; other text messaging services such as Twilio may be a more reliable deployment method.

### Conclusions

In this pilot mHealth intervention, we found text messages to be a feasible method of encouraging avoidance of SHS exposure among adolescents. However, the sample did not demonstrate an overall significant reduction in biochemical exposure.

## Abbreviations

ADHD: attention deficit hyperactivity disorder
BLQ: below the limit of quantitation
CHC: Children’s Health Center
LOQ: limit of quantitation
mHealth: mobile health
SES: socioeconomic status
SHS: secondhand smoke
